# A cooperative effect of ligands, Mg^2+^ ions, and the U6C mutation on the structural dynamics of the SAM-Ⅵ Riboswitch

**DOI:** 10.1016/j.jbc.2026.113211

**Published:** 2026-05-28

**Authors:** Guodong Hu, Chengfei Cai, Jin Qian, Jianzhong Chen

**Affiliations:** 1 Jiangsu Key Laboratory of Intelligent Drug Screening and Repositioning (TZU), School of Information Engineering, Taizhou University, Taizhou, Jiangsu, China; 2 School of Science, Shandong Jiaotong University, Jinan, China

**Keywords:** SAM-Ⅵ riboswitch, Mg^2+^ ions, molecular dynamics simulations, structural cooperativity, U6C mutation

## Abstract

SAM riboswitches, the most abundant type of riboswitch, are classified into eight classes. SAM-VI differs from all other known SAM riboswitch classes by adopting a unique fold and ligand pocket. To elucidate the cooperative structural dynamic effects of ligands, Mg^2+^, and the U6C mutation on SAM-VI, we carried out extensive MD simulations totaling 128 μs. Free energy decomposition identified four key nucleotides (U8, G9, C32, and G33) and suggested a strategy that incorporates entropic contributions for novel ligand design. Approximately eight inner-shell Mg^2+^ ions were predicted, two of which (M3 and M′2) bridge two secondary structures (linking L3-S1 and L2-L1-S2, respectively). In the U6C mutation, M4 and M′8 form inner-shell coordination with the nucleobase of U35, connecting L3 and S3. Bridging Mg^2+^ ions directly alter the RNA secondary structure, and ligand coordination drives additional structural refinement. Collectively, Mg^2+^ ions, ligands, and the U6C mutation exert a hierarchical influence on RNA flexibility: Mg^2+^ and SAM synergistically stabilize the riboswitch, whereas U6C disrupts this stability by elevating regional fluctuation. This work defines the molecular basis of cooperative regulation in SAM-VI, establishing a framework for understanding riboswitch dynamic control and rational ligand design.

Riboswitches, mostly found in bacteria, possess the ability to modulate gene expression by directly sensing cellular metabolites (1). Riboswitches typically reside in the 5′ untranslated regions (UTRs) of mRNAs (2, 3). Riboswitches usually consist of two components—a highly conserved aptamer domain involved in metabolite recognition and a variable expression platform region, which carries gene expression signals. Their gene regulation is directed towards either transcriptional termination or translational repression, both of which exert cis-acting control over metabolite transport and biosynthesis. Since the first description of riboswitches (4), at least 55 validated riboswitch classes for different ligand types have been identified in nature (5).

Adenosylmethionine (SAM; Fig. 1*A*) is an essential metabolite that serves as a methyl donor and transfers the methyl group to substrates (6, 7). In the course of the methylation reaction, SAM donates its methyl group and is converted to S-adenosylhomocysteine (SAH; Fig. 1*A*), an electrically neutral thioether (8). In bacteria, the intracellular concentration of SAM is tightly regulated and frequently modulated by riboswitches. The SAM riboswitches are among the most abundant riboswitches. Based on their structure, sequence, and evolutionary relatedness, SAM riboswitches can be classified into distinct classes, including SAM-I (9), SAM-II (10), SAM-III (11), SAM-IV (12), and SAM-I/IV (13), SAM-V (14), SAM-VI (15), and SAM/SAH (13, 16). In addition to SAM/SAH, all SAM riboswitches exhibit preferential binding towards SAM over SAH (17).

**Figure 1:**
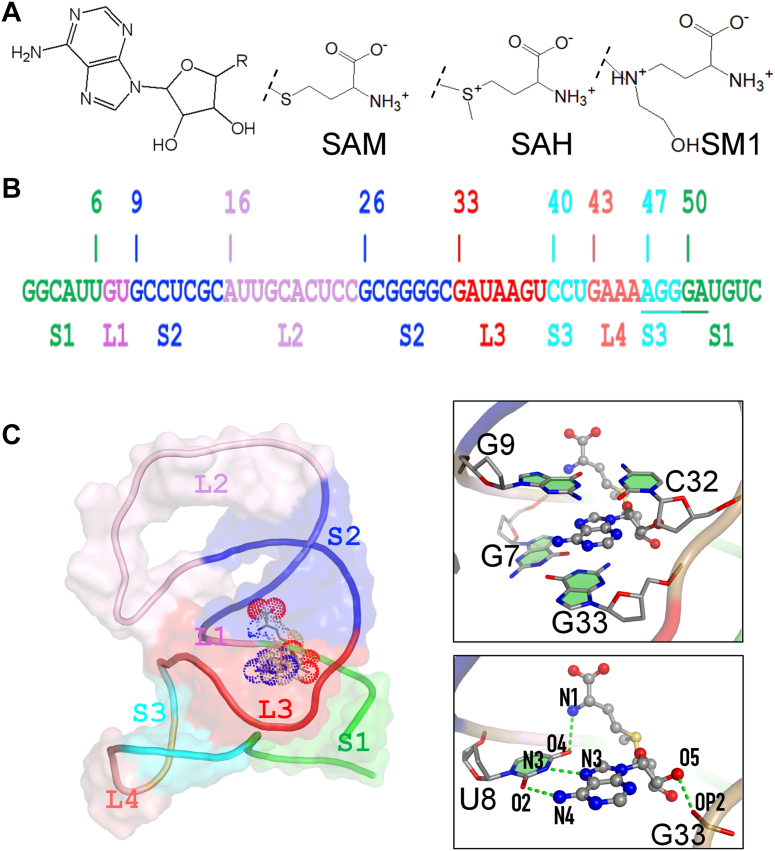
**The ligands, sequence, and structures of the SAM-VI riboswitch.***A*, chemical structures of three ligands. *B*, sequence and secondary structure of the aptamer. *C*, three-dimensional structure of the SAM-VI riboswitch complexed with SAM ligand (PDB ID: 6LAS), with the aptamer shown in both cartoon and surface representation and the ligand SAM shown in both stick and dots representation. Nucleotides in the sequence and in the structure are color-matched. *Right top*: zoomed version showing ligand SAM in binding pocket highlight the base stacking with G9-C32 pair above and with G7-G33 pair below. *Right bottom*: highlighting the hydrogen bonding with U8 and G33. Nucleobases are filled in *green* color.

The SAM-VI riboswitch, functioning as a translational OFF switch, was identified in *Bifidobacterium* species through computational methods of comparative sequence analysis (18). The sequence and three dimensional structure (15) reveal that the SAM-VI riboswitch comprises three stems: Stem S1, formed by base pairing of G1-U6 with C55-G50; stem S2, formed by base pairing of G9-C14 with bases C31 to G26; and a shorter stem consisting of three canonical pairs between C40, C41, and U42 in the 5ʹ strand and G49, G48, and A47 in the 3ʹ strand of a hairpin. The hairpin is not involved in the binding pocket for SAM. A short loop, L1 (G7 and U8) connects the S1 and S2 stems; L2 (A15 to C25) and S2 form a hairpin structure; L3 (G32 to U39) links the S2 and S3. L4 (G44 to A46) forms a hairpin with S3 (Fig. 1, *B* and *C*). A sandwich-shaped ligand-binding pocket is formed between one terminal Watson-Crick base pair G9-C32 from stem S2 and one non-canonical (trans Watson-Crick) base pair G7•G33 (Fig. 1*C*, top right panel). The two pairs stack on the SAM-adenine base from both sides. The SAM is further stabilized by hydrogen bonds: U8 forms a (trans Watson-Crick) base pair with the Hoogsteen edge of the SAM-adenine base; the two Watson-Crick edges of C37 and the SAM-adenine base interact with each other. The 2ʹ-OH of the ribose sugar of SAM forms a hydrogen bond with the non-bridging phosphate oxygen of G33. The methionine tail folds back and forms an H-bond between the α-amino group of the SAM methionine and O4 of U8 (Fig. 1*C*, bottom right panel) (15).

A few SAM/SAH analogs have been synthesized as tools for use in combination with SAM-dependent methyltransferases (19). To utilize the potential alkyl transfer reactivity of SAM analogs in riboswitch-engineered systems, Sun *et al.* (15) synthesized an analog (SM1), wherein the native sulfonium is substituted with a tertiary amino group (Fig. 1*A*). SM1 exhibits the capability to compete with SAM and resembles SAH at higher pH values.

Mg^2+^ ions are essential for RNA tertiary structure folding (20, 21, 22), stabilizing (23), mediating ligand binding (16, 24, 25, 26, 27), and participating in catalysis (28). However, identifying Mg^2+^ ions in RNA structures by experimental approaches is very challenging because the RNA can fold to an ensemble of conformations. Using X-ray diffraction to distinguish Mg^2+^ from Na^+^ and water molecules requires high resolution (29) because they all have the same number of electrons.

Several novel approaches have been developed to predict RNA-metal ion binding sites. These methods can be categorized into knowledge-based approaches and physics-based approaches. Knowledge-based approaches, such as MetalionRNA (30) and Monte Carlo tightly bound ion (MCTBI) (31, 32), rely on physicochemical information derived from experimental structures. Physics-based approaches, including molecular dynamics (MD) simulations, explicitly account for physical energetics and dynamics involved in RNA-ion interactions (33). Molecular dynamics (cMD) simulations have been used to predict or characterize Mg^2+^ binding sites in RNA (34, 35, 36, 37, 38, 39) and the conformational change of protein (40, 41, 42). We have successfully achieved Mg^2+^-RNA inner-shell coordination in MD simulations by predicting the Mg^2+^ binding site on the initial structure used for the simulations (39). MD simulations have demonstrated that Mg^2+^ ions can stabilize the conformation of RNA (37, 43), promote the conformational transition (20), facilitate the RNA-ligand binding (44), influence the pathways of RNA-ligand binding and unbinding (39), and participate in the transmission of ligand binding information (45, 46).

In MD simulations, the force field parameters of Mg^2+^ present challenges in providing a quantitative description (33). Although the parameters developed by Li *et al.* (47) were derived from solution properties, they have been utilized in recent research for the RNA-Mg^2+^ interaction with better performance (44, 45, 46, 48). Panteva *et al.* assessed the ability of 17 different Mg^2+^ ion models to simultaneously reproduce structural, thermodynamic, kinetic and mass transport properties in aqueous solution; their results suggested that the Li model (49) performs extremely well across all properties (50). Furthermore, they developed 12-6-4 potential models for nucleic acid binding (51). Bergonzo *et al.* (20) demonstrated that five models can accurately describe the dynamic structure shift from a magnesium-free to a magnesium-bound conformation in an RNA stem-loop. Additionally, the Aquist (52) and Li (49) parameters effectively describe the RNA–Mg^2+^ interactions *via* first-shell water molecules (20).

In this work, we carried out 128 μs of MD simulations on the SAM-VI riboswitch aptamer (Table 1) to uncover the structural effects of SAM, Mg^2+^, and mutation U6C on the SAM-VI riboswitch aptamer. Four nucleotides (U8, G9, C32, and G33) were identified as crucial for ligand binding. Both the ligands and the U6C mutation influenced the distribution of Mg^2+^ ions, which in turn affected the secondary structure and flexibility of the RNA.

**Table 1:** Summary of molecular dynamics (MD) simulations

Systems	Mg^2+^	Number of replicates	Length of trajectory (μs)	Total time (μs)
Apo, SAM, SAH, SM1, U6C	Yes	8	2	80
Apo, SAM, SAH	No	8	2	48

## Results

MD simulations were employed to investigate the SAM-VI riboswitch aptamer, comparing the apo and SAM-bound states in the presence of Mg^2+^. We organize the results around three central questions prioritized by functional relevance. We first establish a baseline of dynamic behavior by characterizing how Mg^2+^, ligands, and the U6C mutation modulate RNA flexibility, and then map the spatial and functional distribution of Mg^2+^ binding sites within the SAM-VI aptamer. Building upon these foundational insights, we subsequently address the overarching question of how these three factors cooperatively shape SAM-VI’s structure and ligand-binding affinity. This is achieved by detailing the opposing mechanical effects on the binding groove, the architectural roles of specific ion networks, the underlying thermodynamic and microscopic determinants, and ultimately, the electrostatic remodeling driven by ligand binding.

### The effects of Mg^2+^, SAM, and mutation U6C on the flexibility of RNA

**Figure 2:**
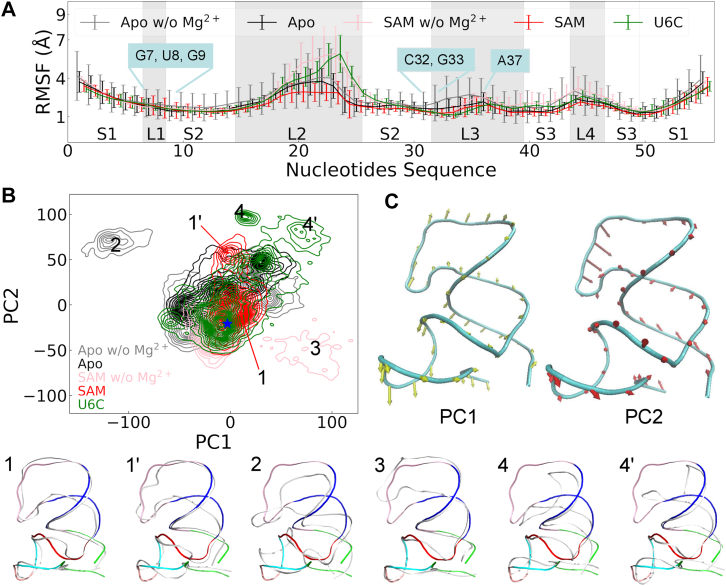
**Effects of Mg^2+^, SAM and U6C mutation on flexibility of RNA.***A*, the RMSFs of P, O3′, O5′, C3′, C4′, and C5′ in the apo form and the SAM-bound with and without Mg^2+^ ions, as well as U6C mutation, each averaged over eight replicate cMD runs. The error bars represent the standard deviations of RMSFs.The key ligand-contacting nucleotides (G7, U8, G9, C32, G33, and A37) are labeled. *B*, density maps generated using the first two principal components (PC1 and PC2). The coordinates of 6LAS are denoted by a *blue star*, while representative structures for each basin are superimposed onto 6LAS in white color. *C*, motions along PC1 and PC2 were analyzed by depicting arrows on the structure to indicate the corresponding PC vectors.

Conformational flexibility can quantify the allosteric effect in the absence of significant conformational changes (53). We quantified flexibility *via* root-mean-square fluctuations (RMSFs) of RNA backbone atoms (P, O3′, O5′, C3′, C4′, and C5′) across all systems (Fig. 2*A*). SAM-VI exhibits inherent rigidity in stem regions and flexibility in loops (L1-L4), with Mg^2+^ as the primary stabilizer—89.1% (apo) and 90.9% (SAM-bound) of nucleotides show reduced RMSFs in the presence of Mg^2+^. Comparing the RMSF values between the apo form and SAM-bound form suggests that the RMSF values of 27 nucleobases are smaller in the SAM-bound form compared to the apo form in the absence of Mg^2+^. However, in the presence of Mg^2+^, this number drops to 6, indicating Mg^2+^ pre-stabilizes the riboswitch, and SAM binding refines this stability rather than driving it. The U6C mutation abrogates this stabilization, increasing fluctuations for 81.8% of nucleotides (notably in L2).

To further evaluate the conformational variations affected by the Mg^2+^, SAM and mutation U6C, the principal component analysis (PCA) was also carried out on the 80,000 snapshots collected from 40 cMD runs for five systems. The variance contribution rates of the first two principal components (PC1 and PC2) are 43.2% and 38.4%, respectively, accounting for a total of 81.6% of the correlated motion space of the SAM-VI riboswitch. The projection of PC1 and PC2 reveals a landscape dominated by a primary free-energy basin (Basin 1), where the density distributions of all five systems significantly overlap (Fig. 2*B*). This clustering indicates that the native fold serves as a global conformational attractor, stabilized by both Mg^2+^ and ligand binding. Notably, structures extracted from Basin 1 and its neighbor (Basin 1′) in the SAM-bound system superimpose well onto the crystal structure, yielding backbone RMSDs of 3.35 Å and 2.91 Å, respectively. In contrast, the absence of Mg^2+^ in the apo state induces a distinct conformational shift to Basin 2, characterized by a significant structural deviation with an RMSD of 7.95 Å. This finding contextualizes the RMSF results, confirming that Mg^2+^ is critical for preventing large-scale unfolding. While the SAM-bound state without Mg^2+^ populates a shallow, distinct basin (RMSD 3.50 Å), indicating increased flexibility. SAM-bound state without Mg^2+^ remains structurally closer to the native state than the apo form, reinforcing the role of SAM in maintaining core rigidity. The U6C mutation, however, induces the most pronounced structural perturbation among the bound states, populating two distinct basins (4 and 4′) with high RMSDs (6.43 and 6.72 Å). These deviations exceed those observed in the SAM-bound state lacking Mg^2+^, highlighting the U6C mutation’s dominant role in destabilizing the L2 region.

The motions along PC1 and PC2 are depicted in Figure 2*C*. PC1 captures the independent motion of L2 from L3 and L4, while PC2 corresponds to a backward swing motion of L2. In the crystal structure, the L2-L3 interface is stabilized by a hydrogen bond formed between the sugar O2′ of C24 and the base N3 of U39. Analysis of the d_O2'-N3_ distance distribution (Fig. S1) reveals that structures within Basin 2 maintain an average distance of 3.43 ± 0.39 Å, indicative of an intact hydrogen bond. Conversely, the global unfolding observed in the Mg^2+^-free apo state correlates with a dramatic elongation of this distance to 6.47 ± 3.79 Å. This causal link establishes that Mg^2+^ ions are essential for preserving the L2-L3 interface, and their absence leads to hydrogen bond disruption and subsequent global structural destabilization.

### How do Mg^2+^ ions bind to the SAM-VI riboswitch aptamer?

Initial Mg^2+^ positioning was added by the MCTBI method (31, 32), which stratifies ions into tightly bound and diffusely bound populations to efficiently sample low-energy states while accounting for ion correlations. The tightly bound ions are treated explicitly through many-body distribution enumeration, whereas diffusely bound ions are modeled implicitly *via* the nonlinear Poisson-Boltzmann equation (31). The three-dimensional interaction site model (3D-RISM) also considered the correlation effect by providing a direct solution to the 3D density distribution around the RNA (54, 55, 56).

To ascertain the spatial distribution of Mg^2+^ ions during the simulation, we calculated the radial distribution function (RDF) of Mg^2+^ around the oxygen and nitrogen atoms of the RNA (Fig. S2). The RDF reveals two distinct coordination shells: an inner-shell peak at 2.0 Å and an outer-shell peak at 4.3 Å. In comparison to the SAH/SAM riboswitch studies (46), the inner-shell coordination of OP1 and OP2 exhibits a 2- to 3-fold unfavorable trend, suggesting a reduced propensity for direct chelation in SAM-VI. Outer-shell coordination is predominantly formed with OP1 and OP2 on the backbone, as well as N3 of uracil bases. Additionally, the pronounced peaks in the RDF for the O2 and O4 atoms of uracil nucleobases strongly suggest the presence of inner-shell coordination. We also noticed an outer-shell peak for the N7 atom of adenine or guanine bases, although the force field underestimates the interaction with the N7 of the purines (50, 51). The coordination statistics derived from our simulations show remarkable agreement with crystallographic survey data (29) and previous riboswitch studies (46).

**Figure 3:**
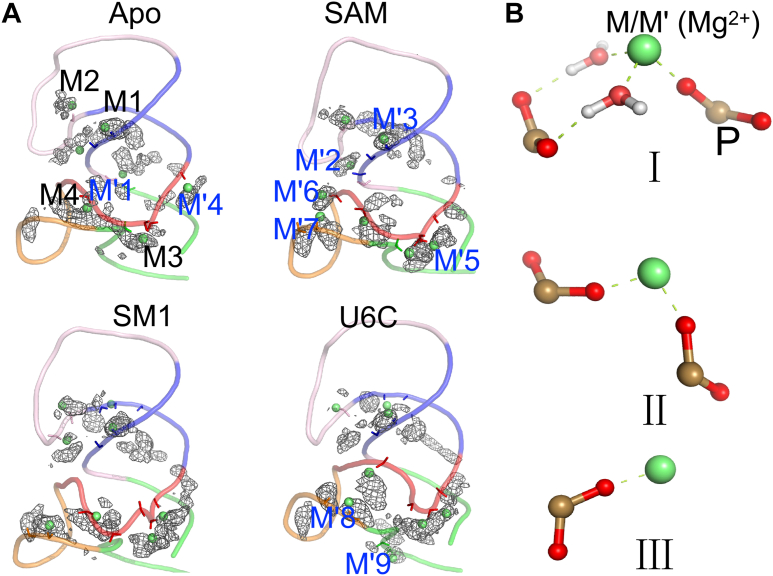
**The distribution of Mg^2+^ ions around RNA during MD simulations and their three models.***A*, a representative snapshot from the MD simulations showing stable Mg^2+^ ions (*green spheres*) coordinated by phosphate groups (sticks). The spatial density of the Mg^2+^ ions is rendered as a grey mesh and superimposed on the structure. Ions that were consistently stabilized in all four simulated systems are labeled M1-M4 (*black*), whereas the remaining stable ions are designated M′1-M′9 (*blue*). *B*, schematic representations of three distinct binding models between Mg^2+^ and phosphate groups. Dashed lines indicate hydrogen bonds.

To determine the precise positioning of each inner-shell Mg^2+^ ion on the RNA, we analyzed the distance distribution between Mg^2+^ and the non-bridging phosphate oxygen atoms (OP1 and OP2). The presence of a distinct peak at approximately 1.97 Å in these distributions signifies a direct interaction or inner-shell coordination of Mg^2+^ with the corresponding phosphate group, while an indirect interaction or outer-shell coordination is indicated by a single peak around 4.6 Å (Figs. S3 and S4). We observed 7, 9, 8, and 8 Mg^2+^ ions forming inner-shell coordination in the apo form, SAM-bound, SM1-bound, and the U6C mutation, respectively. The inner-shell Mg^2+^ ions were conserved across all eight replica simulations. The distance densities for the inner-shell Mg^2+^ in each simulation were similar to the overall average (Figs. S5–S8). The average correlation coefficient between the distance density of any individual simulation and the total exhibited a minimum of 0.90 ± 0.08. Notably, these nucleotides exhibit the most negative electrostatic potential on the surface of the RNA molecule (Fig. S9). The densities of Mg^2+^ ions around the RNA are superimposed on a representative snapshot of inner-shell Mg^2+^ ions (Fig. 3*A*). Four inner-shell Mg^2+^ ions, designated M1 to M4, are conserved in both the apo form and ligand-bound forms (Figs. 3*A* and S3). Additionally, there are inner-shell Mg^2+^ ions found in at least one but not all systems, which we refer to as M′1 to M′9 (Figs. 3*A* and S4). These inner-shell Mg^2+^ ions are predominantly located in loop L3 and the connection region between L2 and S2. In contrast to previous studies (46) on SAH/SAM riboswitches, where conserved Mg^2+^ ions were primarily observed in the major groove, our findings demonstrate that the four conserved Mg^2+^ ions in both apo and bound forms are localized within the minor groove region.

Guided by RDF analysis, we further investigated the specific coordination sites of Mg^2+^ with uracil nucleobases. The inner-shell M4 also forms inner-shell coordination with the O2 atom of U35 in the apo form, SAM-bound, SM1-bound, and U6C mutation (Fig. S10*A*). In contrast, a specific interaction is observed only in the U6C mutant, where the inner-shell M′8 also forms inner-shell coordination with the O4 atom of U35 only in the U6C mutation (Fig. S10*B*).

According to the distances of interaction and whether the interaction is mediated by water molecules, three distinct binding models are proposed and designated as Model Ⅰ, Ⅱ, and Ⅲ (Fig. 3*B*). In Model Ⅰ, inner-shell Mg^2+^ ions also form an outer-shell coordination with an adjacent phosphate group. Model Ⅱ involves Mg^2+^ ions simultaneously forming inner-shell coordination with two adjacent phosphate groups in a bidentate configuration (57). In Model Ⅲ, the interaction of inner-shell Mg^2+^ ions is limited to a single phosphate group. Model Ⅰ is the most favorable, while Model Ⅱ is exclusively observed in SM1-bound for M′5 with nucleotides G33 and A34 (Figs. S3 and S4). This classification framework provides a structural basis for understanding how specific binding geometries contribute differentially to RNA stability.

### Ligand and Mg^2+^ exert opposing effects on the dynamic expansion of the binding groove

**Figure 4:**
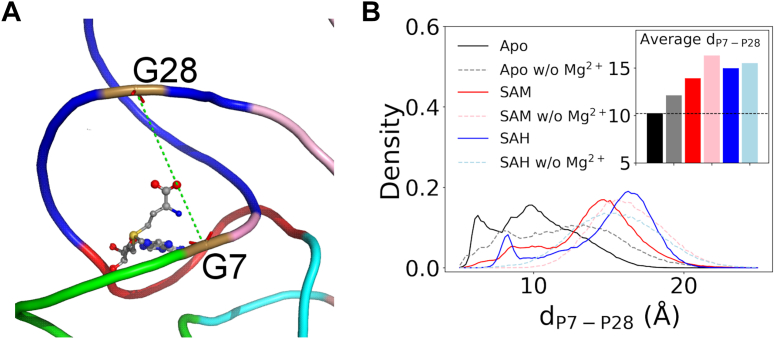
**The changes in three-dimensional structures by Mg^2+^ ions and ligand.***A*, the distance d_P7-P28_ between the phosphorus atoms of G7 and G28 for measuring the groove width. *B*, distributions of d_P7-P28_ in the simulations of the apo forms SAM-bound and SAH bound without (labeled as “w/o”) or with saturating Mg^2+^. Inset: average values for six systems.

The 5′-deoxyadenosyl groups of the ligands insert deeply into the binding pocket (Fig. 1*C*), while the aminocarboxypropyl groups alternate between "U"-shaped and "T"-shaped conformations within a spacious groove defined by nucleotides 4 to 8 and 27 to 31 of the RNA (Figs. 1*C* and 4). To quantify the dynamic response of this groove, we measured the distance (d_P7-P28_) between the phosphorus atoms of G7 and the G28 (Fig. 4*A*). Analysis reveals two opposing conformational drivers: ligand binding induces a pronounced groove expansion of ∼ 4 Å (under both saturating and Mg^2+^-free conditions), whereas Mg^2+^ saturation counters this expansion, reducing the width by ∼ 2.0 Å in the apo and SAM-bound forms and by 0.54 Å in the SAH-bound form (Fig. 4*B*).

Compared to the study on the SAM/SAH riboswitch (46), both SAM/SAH and SAM-VI riboswitches exhibit expansion of the major groove upon ligand binding; however, the groove shrinks upon Mg^2+^ saturation in the SAM-VI riboswitch, while it expands in the SAM/SAH riboswitch. This discrepancy arises from differences in Mg^2+^ distribution. In the SAM-VI riboswitch, unstable Mg^2+^ ions are situated within the central groove, exerting an attractive force on the phosphate groups on both sides. Conversely, in the SAM/SAH riboswitch, six fixed Mg^2+^ ions are positioned on either side of the groove and exert electrostatic repulsion. Thus, the functional role of Mg^2+^ is context-dependent: in SAM-VI, it acts as a structural brace to contract the groove, whereas in SAM/SAH riboswitches, it functions as a structural wedge to enforce expansion.

### Distinct roles of M3, M′2, and a dual-ion network in stabilizing the RNA structures

**Figure 5:**
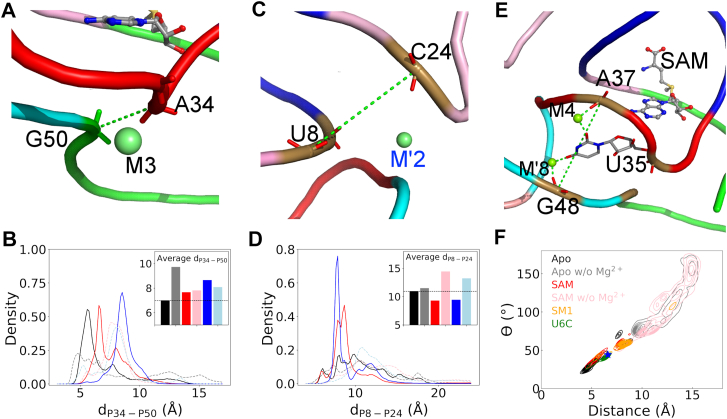
**The changes of three-dimensional structure caused by Mg^2+^ ions.***A*, the distance, d_P34-P50_, between two phosphorus atoms of A34 and G50 for measuring the interaction between L3 and S1. *B*, Distributions of d_P34-P50_, in the simulations of the six systems. Inset: average values. *C*, The distance, d_P8-P24_, between two phosphorus atoms of U8 and C24 for measuring the interaction between L1 and L2. *D*, Distributions of d_P8-P24_ in the simulations of the six systems. Inset: average values. The panels *A* and *C* represent a zoom view of Figure 1*C*, left; the d_P34-P50_ and d_P8-P24_ shown in *green* dash lines. *E*, the distance, d_P37-P48_, between two phosphorus atoms of A37 and G48; the distance, d_O2-M'8_, between M4 and O2 of U35; the distance, d_O2-M4_, between M′8 and O4 of U35. *F*, density maps in the space of two collective variables: the distance between the center of two phosphorus atoms of A37 and G48 and the center of the nucleobase of U35, and the angle (Ɵ) formed by the phosphorus atom of G38, phosphorus atom of A36, and the center of the nucleobase of U35. The experimental structure of the SAM-bound state (PDB ID: 6LAS) is marked by a *blue star*.

Mg^2+^ ions that bridge distinct secondary structure elements play a pivotal role in global architecture compared to those coordinating adjacent nucleotides. We specifically investigated M3 and M′2, which interact with phosphate groups from different region of secondary structures play a more crucial role in stabilizing the RNA structure compared to Mg^2+^ ions that connect adjacent nucleotides. M3 in Model Ⅰ establishes an inner-shell coordination with A34 and outer-shell coordination with both U35 and G50, connecting L3 and S1 in both the apo form and SAM-bound (Figs. 5*A* and S3). To quantify the structural impact of M3, we monitored the inter-phosphate distance d_P34-P50_ (Figs. 5, *A* and *B*). In the apo form, Mg^2+^ saturation induces a significant compaction, reducing d_P34-P50_ from 9.7 Å to 7.0 Å, while SAM-binding partially resists this contraction (decreasing only 0.2 Å), the absence of M3 in the SAH-bound form results in a pronounced increase in distance. These findings delineate a competitive mechanism: M3 functions as a structural clamp to tighten the L3-S1 interface, a stabilizing force that is counteracted by the steric occupancy of the ligand binding pocket.

Simultaneously, M′2 (Model III) anchors L2, L1, and S2 by interacting with C24 and forming outer-shell contacts with U8 and G9 (Figs. 5*C* and S4). The distribution of the distance d_P8-P24_ (Figs. 5, *C*, *D* and S10*C*) reveals that M′2 is essential for maintaining the L2-L1 interface. In the presence of M′2 (SAM-, SAH-, and SM1-bound), d_P8-P24_ exhibits a sharp distribution centered at ∼8.0 Å, representing a contraction of 3.8 to 5.1 Å compared to Mg^2+^-free states. The functional necessity of M′2 is underscored by the U6C mutant: despite saturating Mg^2+^ concentrations, the absence of M′2 results in a destabilized, elongated conformation (d_P8-P24_≈13.2 Å), closely mirroring the disordered states of Mg^2+^-free systems. Thus, M′2 acts as a critical anchor, restraining L2 at the apex of the binding pocket.

The nucleobase U35 serves as a central hub for ion-mediated stabilization, coordinating with M4 and, specifically in the U6C mutant, M′8 (Figs. 5*E* and S10, *A*, *B*). While M4 is conserved across systems, the U6C mutant exhibits a uniquely stable coordination network. The density distribution of d_P37-P48_ reveals that only U6C maintains a stable interface between A37 (L3) and G48 (S3) (Fig. S10*D*). This stability arises from a dual-ion bridge where U35 simultaneously coordinates M4 and M′8; in systems lacking M′8, this stable L3-S3 interaction is disrupted.

This network governs the conformational switching of U35. The nucleobase of U35 in the experimental structures of SAM-bound (PDB ID: 6LAS) and SAH-bound (PDB ID: 6LAU) adopt an "in" conformation by inserting themselves into the groove between L3 and S3 (Fig. S11*A*). In contrast, in the structures of SM1-bound (PDB ID: 6LAZ) and U6C mutation (PDB ID: 6LAX), the nucleobase of U35 is exposed to the solvent, adopting an "out" conformation (Fig. S11*B*). To gain a global understanding of the conformational space, we monitored two parameters (1): the distance between the centers of the phosphorus atoms of A37 and G48 and the center of the nucleobase of U35, and (2) the angle (Ɵ) formed by the phosphorus atoms of G38 and A36, and the center of the nucleobase of U35. The density maps of the distance and the angle are present in Figure 5*F*. The density map of U6C reveals a small, concentrated basin that includes a peak corresponding to the angle and distance values of the experimental structure of SAM-bound (PDB ID: 6LAS). The density maps of the other three systems containing M4 also exhibit smaller basins compared to those of systems without M4. Notably, while the starting structures of SAM-bound with and without Mg^2+^ are identical, their respective density maps show significant differences. The nucleobase of U35 initially inserts into the RNA in an “in” conformation in the starting structure and maintains this conformation throughout the simulation when the Mg^2+^ ions are present (Fig. S11*A*; Movie S1). However, following equilibration in simulations without Mg^2+^, the nucleobase of U35 becomes exposed to the solvent in an “out” conformation in both the SAM-bound and apo forms (Fig. S11*B*; Movie S2).

### Energetic decomposition reveals the thermodynamic impact of ligand binding and the U6C mutation

Quantitatively replicating experimental data through computational methods poses significant challenges, particularly for calculating the absolute binding free energy. However, the relative binding free energy between different ligands to same aptamer can be very informative (58). Compared to the MM-PBSA method, the MM-GBSA produces better performance in ranking the binding affinities for systems without metals (58). To explain the disparities in binding affinities between the cognate (SAM) and synthetic (SM1) ligands, as well as wild-type SAM-VI aptamer (WT) and its U6C mutation (U6C), we calculated the binding free energies by using the MM-GBSA method (Table 2). The MM-GBSA has been successfully used on many RNA-ligand (44, 59) and protein-ligand (60, 61) systems.

**Table 2:** Binding free energies and their components (in kcal/mol) for five ligands^a^

Components	WT-SAH	WT-SAM	WT-SM1	U6C-SAM
$\Delta E_{\text{ele}}$	−70.99 (1.77)	−1183.07 (5.81)	−1210.76 (2.11)	−1203.3 (14.75)
$\Delta E_{\text{vdW}}$	−41.22 (0.40)	−43.86 (0.61)	−47.62 (0.63)	−46.41 (1.42)
$\Delta G_{\text{pol}}$	88.23 (1.52)	1186.27 (6.11)	1217.81 (1.71)	1208.0 (15.32)
$\Delta G_{\text{nonpol}}$	−2.46 (0.04)	−2.62 (0.06)	−3.03 (0.05)	−2.81 (0.10)
$\Delta E_{\text{ele}}+\Delta G_{\text{pol}}$	17.24 (0.49)	3.20 (0.42)	7.05 (0.74)	4.70 (0.60)
$\Delta E_{\text{vdW}}+\Delta G_{\text{nonpol}}$	−43.68 (0.43)	−46.48 (0.65)	−50.65 (0.64)	−49.22 (1.51)
ΔH	−26.44 (0.51)	−43.28 (0.53)	−43.60 (0.90)	−44.52 (0.92)
TΔS	−20.02 (0.43)	−21.61 (0.50)	−24.71 (0.54)	−22.47 (0.69)
$\Delta G_{\text{bind}}$	−6.42 (0.46)	−21.67 (0.58)	−18.89 (0.78)	−22.05 (0.44)
$\Delta G_{\exp}$^b^	−6.7	−8.7	−7.2	−8.5

a Standard error of the mean for a sample of eight points in a parenthesis.

b Calculated from the experimental dissociation constants (K_D_) (15) according to $\Delta G_{\exp}=\text{RT}\ln\;K_{D}$, where R is the gas constant, and T is 293K using a solvent of 40 mM HEPES (pH 7.0), 50 mM KCl, and 10 mM MgCl_2_.

The MM-GBSA predicted absolute binding free energy for the SAH-bound is consistent with the experimental data (15); however, the remaining predicted absolute binding free energies exhibit significantly higher values compared to their corresponding experimental counterparts. We noted that the ligands except SAH are charged (Fig. 1*A*). This may further reflect the limitations of the MM/GBSA in estimation of binding free energies of highly polar or charged molecules (62). The results indicate that the ligand SM1 exhibits weaker binding affinity compared to SAM, surpassing the margin of error.

Comparing with the SAH-bound, both the SAM-bound and SM1-bound significantly contribute to the electrostatic component ($\Delta E_{\text{ele}}$), which is largely offset by the polar solvation component ($\Delta G_{\text{pol}}$). Consequently, the polar components ($\Delta E_{\text{ele}}+\Delta G_{\text{pol}}$) for both SAM-bound and SM1-bound exhibit a favorable contribution (by ∼12 kcal/mol) compared to the SAH-bound. The binding of SM1 is energetically less favorable compared to SAM, with a difference of 3.85 kcal/mol for the polar components ($\Delta E_{\text{ele}}+\Delta G_{\text{pol}}$). However, this disparity is counterbalanced by $\Delta E_{\text{vdW}}+\Delta G_{\text{nonpol}}$ offsets (3.17 kcal/mol). These contrasts can be easily attributed to the long-range electrostatic attraction and the bulkier sizes of the ligand SM1. The binding affinities for ligand SM1 are 13-fold lower compared to SAM, with a K_d_ of 4.4 μM. The enthalpic contribution (ΔH) of the ligand SAM is nearly equivalent to that of the ligand SM1, with a negligible difference of 0.32 kcal/mol within statistical error; however, their entropic contributions exhibit a substantial disparity of 3.10 kcal/mol. It is a sound strategy to consider the entropic effect when designing novel potent ligands.

The U6C mutation results in an unfavorable polar component ($\Delta E_{\text{ele}}+\Delta G_{\text{pol}}$) of 1.3 kcal/mol, but a favorable non-polar component ($\Delta E_{\text{vdW}}+\Delta G_{\text{nonpol}}$) of 2.74 kcal/mol. The entropic contribution is attenuated by the favorable enthalpic contribution (ΔH). The predicted total binding free energy ($\Delta G_{\text{bind}}$) for the SAM-bound U6C mutation is slightly more favorable than that of the WT SAM-bound, and this difference falls within the margin of error. This suggests that the U6C mutation alters the local electrostatic environment without significantly compromising the global thermodynamic stability of the complex.

### Nucleotide-ligand interaction energies reveal the key nucleotides

**Figure 6:**
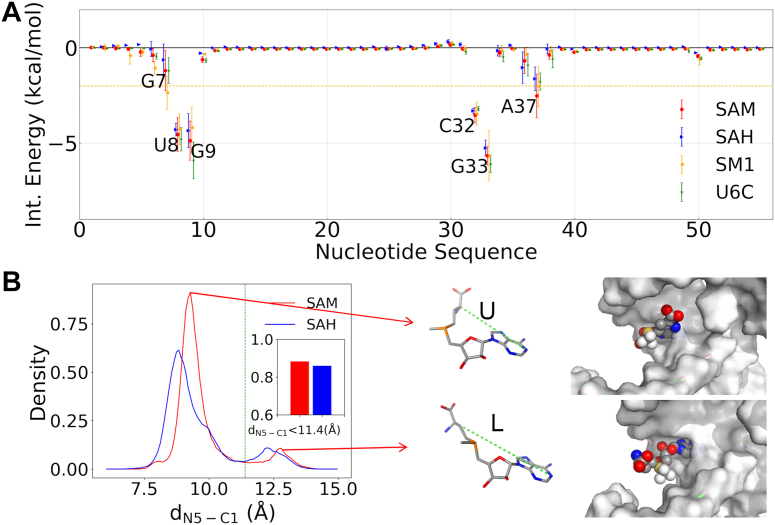
**Interaction between ligands and aptamer.***A*, interaction energies of individual nucleotides with the ligands. The symbol and error bar represent the mean and standard deviation, respectively. The key nucleotides are separated out by a dashed horizontal orange line at −2.0 kcal/mol. *B*, *Left*: distributions of the distance, d_N5-C1_, which is between the N5 atom of adenine base and N1 atom of aminocarboxypropyl in SAM and SAH ligands. Inset: fraction of frames for d_N5-C1_ < 11.4 Å, which has the minimum density between the first and second peaks for SAM. Middle: U-shape and L-shape conformation of SAM. *Right*: SAM in both U-shape and L-shape conformations in the binding pocket, illustrated by a snapshot from the MD simulations.

The interaction energies of the 55 nucleotides of the SAM-VI riboswitch aptamer with ligands by the MM/GBSA method (61) were calculated to identify the key nucleotides with major contributions to the total ligand binding energy (Fig. 6*A*). Among the six key nucleotides, four exhibit significant contributions to the binding energy exceeding 2 kcal/mol in all four complexes. Additionally, two nucleotides (G7 and A37) demonstrate substantial contributions in at least one of the complexes. Although the methyl of SAM does not directly interact with specific nucleotides, all key nucleotides exhibit a preference for SAM binding, which can be attributed to long-range electrostatic attraction between the positive charge on its sulfur center and the RNA phosphates, a feature absent in SAH (Fig. 1*A*).

Despite sharing an identical positive charge with SAM, the analog SM1 exhibits weakened interactions at critical sites. Specifically, U8, G9, and A37 show less favorable contributions in the SM1-bound state compared to SAM. Additionally, the contribution of G7 in SM1-bound is approximately 2 to 4 times greater than that in the SAM-bound or SAH-bound. The crystallographic structures suggested a hydrogen bond and a pairing interaction between U8 and ligands (Fig. 1*C*, right bottom). The hydrogen bond between the atom O4 of U8 and the atom N4 of α-amino group is found in 60.9% snapshots in SAM-bound, but decreases to 55.8% and 54.3% in SM1-bound and SAH-bound, respectively. In the pairing interaction, the U8 nucleobase forms two hydrogen bonds with the SAM-adenine. The hydrogen bond between O2 of U8 and N4 of SAM is present in 44.5% of frames, which represents a higher occurrence compared to its presence in 26.4% of frames in the SM1-bound form. Additionally, the other hydrogen bond formed by two N3 atoms of U8 and SAM/SM1 exhibits a slightly reduced frequency, occurring in 95.1% of frames in the SAM-bound compared to 92.8% in the SM1-bound. The base stacking drives the interaction between the G9 nucleobase and the adenine of ligands. To compare the base stacking in SAM-bound and SM1-bound, we measured the distance between two base centers and the overlapped area of two bases in one plane (44, 45). We observed a slight increase in the distance from 4.7 Å in SAM-bound to 4.8 Å in SM1-bound, while the area decreased from 1.06 to 1.02 Å^2^, indicating a weaker binding affinity for G9 in SM1-bound compared to in SAM-bound. A trans Watson-Crick pair is formed by two purines, A37 and SAM/SM1, with hydrogen bonds observed between the 6-amine of A37 and the 1-nitrogen atom of SAM in 59.1% and 49.0% of snapshots for SAM-bound and SM1-bound, respectively. Additionally, the other hydrogen bonds between the 1-nitrogen atom of A37 and the 6-amine of SAM are present in 82.6% and 76.6% snapshots for SAM-bound and SM1-bound states, respectively.

In the MD simulations, both ligands SAM and SAH exhibit two distinct shapes, namely the "U" and "L" shapes (Fig. 6*B*). Specifically, in 88.2% of the simulated structures, SAM adopts a U-shape configuration with its methyl group predominantly exposed to the solvent; similarly, in 85.8% of the structures, SAH assumes a U-shape conformation as well. The L-shaped conformations are relatively rare and also extend the aminocarboxypropyl group into the solvent. The methyl of SAM does not directly interact with specific nucleotide in both "U" and "L" shapes.

### Ligands binding remodels the local electrostatic environment to recruit specific Mg^2+^

**Figure 7:**
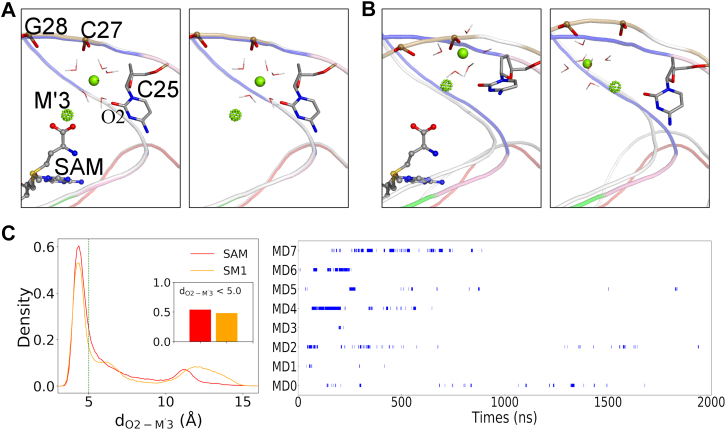
**A rearrangement of the inner-shell Mg^2+^ ions caused by Ligand binding.***A*, the start and end sites of M′3 during the minimization stage shown in dots and surface representation, respectively. Five water molecules within 3 Å of M′3 in the end structures shown in lines. The O2 atom of C25 out-shell coordinate with M′3 is labeled. Left: SAM-bound; Right: apo form. *B*, at the heating stage, the sites of M′3 for SAM-bound (*left panel*) and apo form (*right panel*), the start and end sites of M′3 shown in dots and surface representation, respectively. *C*, *Left*: Distributions of d_O2-M'3_, which is from the O2 atom of nucleotide C25 and M′3. Inset: fraction of frames with d_O2-M'3_ less than 5.0 Å. *Right*: the inner-shell coordination formed across all eight MD simulation replicas.

Ligand binding actively modulates the local distribution of Mg^2+^ ions, as evidenced by the divergent trajectories of ions near the binding pocket. While the apo and SAM-bound systems initiated from identical coordinates, the ion designated M′3 followed distinct pathways during equilibration (Movies S3 and S4). During the minimization stages of SAM-bound and apo form, the Mg^2+^ ions migrate to a similar position, establishing the outer-shell coordination with both the O2 atom of nucleobase C25 and a non-bridging phosphate oxygen of C27 in both simulations (Fig. 7*A*). During the heating stage, in the SAM-bound simulation (Fig. 7*B*, left panel), the Mg^2+^ ion undergoes a rearrangement of water molecules and migrates to a non-bridging phosphate oxygen of C27, forming an inner-shell coordination as M′3; however, in the apo form simulation (Fig. 7*B*, right panel), the Mg^2+^ ion coordinates with six water molecules. Hence, the ligand facilitates the capture of M′3.

In the SAM-bound simulation, the M′3 forms outer-shell coordination with the oxygen atom of nucleobase C25 in approximately 53.7% of frames, as well as in around 47.8% of frames in the SM1-bound simulation (Fig. 7*C*, right), suggesting a potential weak hydrogen bond between the amino group of C25 and the carboxyl moiety of ligand SAM. Remarkably, this outer-shell coordination was observed across all eight MD simulation replicas (Fig. 7*C*, left), further indicating that the simulations are convergent. Furthermore, ligand binding stabilizes the coordination environment of M′5 (Model Ⅰ). Free energy decomposition analysis suggests that G33 contributes significantly to ligand binding through base stacking and a stable hydrogen bond (Figs. 1*C* and S6*A*). The root mean square fluctuation (RMSF) of G33 exhibits higher values in the apo form (1.70 ± 0.68 Å) compared to the SAM-bound form (1.34 ± 0.48 Å), indicating that G33 exhibits greater stability when bound to a ligand. Thus, ligand binding rigidifies G33, enabling it to collaborate with A34 to effectively capture and retain M′5. These findings illustrate a synergistic relationship where ligands not only occupy the binding site but also actively organize the local electrostatic environment to secure specific Mg^2+^ interactions.

## Discussion

We performed extensive 128 μs molecular dynamics simulations to comprehensively investigate how ligand binding, Mg^2+^, and the U6C mutation cooperatively govern the stability and ligand recognition of the SAM-VI riboswitch aptamer. We synthesize our core findings into a hierarchical regulatory model that defines the functional interdependencies of the three factors: Mg^2+^ acts as the primary structural stabilizer, ligands act as secondary allosteric refiners, and the U6C mutation acts as a selective structural disrupter that preserves core ligand binding.

Our data establish Mg^2+^ ions as the primary structural regulators of SAM-VI riboswitch folding and dynamics. Mg^2+^ ions (≈8 inner-shell) bind SAM-VI *via* three structural models, with bridging Mg^2+^ ions (M3, M′2, M4/M′8) acting as the molecular glue of tertiary structure—linking non-adjacent secondary structures (L3-S1, L2-L1-S2, L3-S3) and reducing RNA flexibility. Conserved Mg^2+^ ions (M1-M4) localize to the minor groove, while system-specific ions (M′1-M′9) enrich in loop regions and mediate ligand/mutation-specific effects. Mg^2+^ also contracts the ligand-adjacent groove *via* electrostatic attraction of flanking phosphates opposing the groove expansion observed in SAM/SAH riboswitches, a structural adaptation shaped by differential Mg^2+^ distribution and the compacted SAM-VI conformation (which harbors 3 fewer inner-shell Mg^2+^ ions than related SAM riboswitches, totaling 8 *versus* 11) (46).

Ligands (SAM, SAH, and SM1) act as downstream allosteric effectors that refine the Mg^2+^ constrained riboswitch conformation. Specifically, ligands expand the Mg^2+^-contracted groove to accommodate the aminocarboxypropyl tail and drive the formation of system-specific Mg^2+^ binding sites (M′3, M′5) that stabilize the ligand-binding pocket. Critically, entropy emerges as the primary determinant of SAM *versus* SM1 ligand specificity in SAM-VI.

The U6C mutation acts as a selective disrupter of the Mg^2+^-ligand regulatory network, triggering structural remodeling while preserving core ligand recognition. Mechanistically, the U6C mutation abrogates Mg^2+^-SAM stabilization, losing M′2 (the L2-L1-S2 bridge), leading to severe L2 flexibility, gaining M′8/U35 inner-shell coordination, creating a unique L3-S3 bridge and driving U35 to an out (solvent-exposed) conformation, and increasing global RNA fluctuation for 81.8% of nucleotides. Surprisingly, the mutation has no significant effect on SAM binding affinity—due to the robustness of the core four ligand-binding nucleotides. This interaction network insulates ligand recognition from global structural perturbations, an evolutionary adaptation likely enabling SAM-VI to tolerate minor sequence variations in *Bifidobacterium* hosts.

Our computational work delivers two transformative advances: a hierarchical model for structural regulation and an entropy-guided framework for rational design. To validate the predicted synergistic stabilization by Mg^2+^ and SAM, as well as the disruptive flexibility caused by the U6C mutation, we propose a multi-faceted biophysical approach. First, Fluorescence Resonance Energy Transfer (FRET) will serve as the gold standard for quantifying RNA conformational dynamics. By site-specifically labeling SAM-VI at L2 (C24) and L3 (U39) to monitor L2-L3 motion, and at S1 (G1) and L3 (A34) to probe the M3 bridge, we can directly measure distance changes. Second, ^1^H-^15^N HSQC NMR will identify Mg^2+^ binding sites with Chemical shift perturbations in key nucleotides (*e.g.*, A34 for M3, C24 for M′2, U35 for M4/M′8), validating predicted bridging interactions. Finally, ITC will demonstrate that single and double mutants exhibit a significant reduction (≥10-fold) in SAM binding affinity, validating our per-nucleotide interaction energy findings. To design novel, high-affinity ligands, we propose an entropically informed design strategy that minimizes ligand flexibility and optimizes hydrophobic contributions while preserving core nucleotide interactions to maintain the enthalpic foundation. Additionally, Mg^2+^ bridging sites serve as precise targets for engineering SAM-VI variants with tunable flexibility and responsiveness.

## Experimental procedures

### Preparation of molecular systems

The starting structures of SAM-bound, SAH-bound, and SM1-bound of SAM-VI riboswitch were from the chain A of the crystal structures 6LAS, 6LAU, and 6LAZ, respectively (15). The U6C is the SAM-bound with a mutation U6C (PDB ID: 6LAX). The SAM was removed from the SAM-bound to generate the apo form. The missing hydrogen atoms on the RNA molecule were added by using the Leap module in AMBER18 (63). The four starting structures are almost the same. Thirty-7 Mg^2+^ ions were added at the sites predicted by the MCTBI method (32). The RNA and Mg^2+^ were placed in a truncated octahedron periodic box of TIP3P (64), with approximately 11,340 water molecules. Excess positive charge was neutralized by Cl^-^ ions. The systems without Mg^2+^ were neutralized by Na ^+^ ions. The distance from the RNA molecule to the edge of the box was set to 12 Å.

The bulk Mg^2+^ concentration in the simulation box is ∼180 mM, deliberately exceeding physiological levels. While Manning counterion condensation theory (65) predicts high local Mg^2+^ concentrations near the RNA backbone to screen its negative charge, using a high bulk concentration bypasses the entropic cost of ion condensation. We adopt this compromise, common in explicit-solvent RNA-Mg^2+^ MD simulations (37, 44, 46, 48), to overcome sampling limitations and ensure the convergence of stable inner-shell Mg^2+^-RNA coordination sites within feasible timescales. Additionally, in the systems with Mg^2+^ that exclude Na^+^ ions, relying on Mg^2+^ for charge neutralization. Although experimental buffers contain monovalent ions, removing Na^+^ is a controlled simplification. We acknowledge that this significantly alters the solvent environment from experimental conditions, but it is essential for dissecting the individual and cooperative effects of Mg^2+^, ligands, and the U6C mutation. In Mg^2+^ free systems, Na^+^ provides charge neutralization and non-specific screening.

The force field parameters for RNA were derived from an improved AMBER ff99 force field (66, 67), while those for Mg^2+^ ions were obtained from Li *et al.* (47, 49). The TIP3P water and Cl-ions were assigned parameters based on Joung and Cheatham (68). The ligand structures were optimized using the Gaussian16 program (69) at the HF/6-31G∗ level. The general AMBER force field (GAFF) (70) was used to generate force field parameters for the ligands with the atomic partial charges assigned using the restrained electrostatic potential (RESP) method (71).

### Conventional molecular dynamics simulations

All energy minimizations and MD simulations were performed using the AMBER18 package (63). To eliminate steric clashes, the starting structure was subjected to energy minimization, first with 2500 steps of the steepest-descent followed by 2500 steps of conjugate-gradient. The system was then gradually heated from 100 K to 300 K in a 50 ps simulation at constant volume with harmonic restraints of 5 kcal/(mol·Å^2^) applied to the RNA and ligand atoms. This was followed by a 50 ps equilibration at constant volume and 300 K, and a 50 ps equilibration at constant pressure at 1.0 atm, with the same restraints in place. An unrestrained equilibration was then performed for 1 ns at constant temperature (300 K) and pressure (1.0 atm). The temperature was regulated at 300 K by the Langevin thermostat (72). The pressure was regulated at 1.0 atm by the Berendsen barostat (73). Bonds involving hydrogen atoms were constrained with the SHAKE algorithm (74), allowing a 2 fs time step. The particle mesh Ewald method (75) was used to treat the long-range electrostatic interactions with a direct-space cutoff of 12 Å. Four replicate production simulations were run for 1 μs at constant temperature and pressure. Coordinates were saved every 100 ps for subsequent analysis.

### Decomposition of binding free energy per nucleotide

The binding free energies between aptamer and ligand and the decomposition into contributions of individual nucleotides were obtained by the MM-GBSA method (76, 77). The total binding free energy ($\Delta G_{\text{bind}}$) is decomposed into five terms:

$$\Delta G_{\text{bind}}=\Delta E_{\text{ele}}+\Delta E_{\text{vdW}}+\Delta G_{\text{pol}}+\Delta G_{\text{nonpol}}-T\Delta S$$

where “Δ” means the difference between the complex and the separated RNA and ligand. $\Delta E_{\text{ele}}$ and $\Delta E_{\text{vdW}}$ represent the average electrostatic interaction energy and van der Waals interactions between the RNA and the ligand in the gas phase. $\Delta G_{\text{pol}}$ was calculated with a modified generalized Born (GB) models (78) at 0 salt concentration. $\Delta G_{\text{nonpol}}$ was estimated from the solvent-accessible surface area (SASA) using the ICOSA method as γSASA+β (79), where the surface tension constant γ and the correction constant β were 0.005 kcal/mol·Å^2^ and 0.0 kcal/mol, respectively. ΔS is the change in entropy upon binding; and T is the absolute temperature. Entropies were obtained using the *nmode* module of AMBER18 (63), with more detailed calculation procedures described in our previous work (44).

The calculation of free energy between ligand and individual nucleotide was performed only for the enthalpic components (ΔE_ele_, ΔE_vdW_, ΔG_pol_, and ΔG_nonpol_). For each complex, a total of 40,000 snapshots were extracted from eight replicate simulations to calculate the total binding free energies and the decomposition per individual nucleotide. The end energies were averaged over 8 replicates.

### Other analyses

All structural analyses were performed on 20,000 frames extracted from the full 2000 ns trajectory of each replicate simulation. The densities of Mg^2+^ were determined using a Python script that imports the MDAnalysis package (80). The CPPTRAJ program (81) was employed for the computation of distances, RMSFs, RDFs, PCA, and hydrogen bonds. The structures were initially aligned using the backbone atoms (P, O3′, O5', C3′, C4', C5′) of the riboswitch to generate an average structure. Subsequently, the deviations of the backbone atoms from the average structure were calculated to determine the RMSF. The RDFs for Mg^2+^ ions were calculated by determining their density in 0.05 Å distance bins from RNA atoms and normalizing by the expected density for a uniform distribution. Hydrogen bonds were defined as a donor-acceptor distance < 3.5 Å and a donor-H-acceptor angle > 120°.

### Statistics and reproducibility

For each system, we carried out eight replicate MD simulations. The mean and standard deviation were calculated using the replicate simulations. We also tested the convergence of the MD simulations by comparing the results calculated in 500 ns blocks along the trajectories for RMSF (Fig. S12).

### Data availability

All data generated or analyzed during this study are included in this published article (and its supplementary data files). The source data for all the plots presented in figures and force field parameters for the ligands are deposited in GitHub at https://github.com/xzszhgd/SAM-VI-riboswitch.

## Code availability

The computational methods are described in the Computational Methods section. All software used is cited in the references and is publicly available. Input files, initial coordinates, and final structures for the MD simulations of the SAM-VI riboswitch (apo form, three ligand-bound states, and the U6C mutant) have been deposited in the GitHub repository: https://github.com/xzszhgd/SAM-VI-riboswitch.

## Supporting information

This article contains supporting information.

## Conflict of interest

The authors declare that they have no conflicts of interest with the contents of this article.
