# Supplementary material for: A cooperative effect of ligands, Mg2+ ions, and the U6C mutation on the structural dynamics of the SAM-Ⅵ Riboswitch
Source: J Biol Chem. 2026 May 28;302(7):113211. doi: 10.1016/j.jbc.2026.113211 (PMC13314739; doi:10.1016/j.jbc.2026.113211)
Supplement: Supporting Figures [file mmc1.docx]

A Cooperative Effect of Ligands, Mg^2+^ Ions, and the U6C Mutation on the Structural Dynamics of the SAM-Ⅵ Riboswitch

Guodong Hu^1,^*, Chengfei Cai^1^, Jin Qian^1^, Jianzhong Chen^2,^*

^1^Jiangsu Key Laboratory of Intelligent Drug Screening and Repositioning (TZU), School of Information Engineering, Taizhou University, Taizhou, Jiangsu, 225300, China,

^2^School of Science, Shandong Jiaotong University, Jinan, 250357, China

*E-mail: [hugd@tzu.edu.cn](mailto:hugd@dzu.edu.cn) or [xzszhgd@163.com (G. H.);](mailto:xzszhgd@163.com;) jzchen@sdjtu.edu.cn or chenjianzhong1970@163.com (J.C.)

Supplementary Information

Legends for Movies

**Movie S1.** Dynamics of the U35 in the “in” conformation (nucleobase oriented toward the RNA core) in the SAM-bound complex with Mg2+ ions. The distance between the phosphorus atoms of A37 and G48 is indicated by a green dash line. This distance reflects the interaction between A37 and G48 mediated by two inner-shell-coordinated Mg2+ ions and the nucleobase of U35. The distance between the midpoint of the two phosphorus atoms and the center of the U35 nucleobase is also indicated by a green dash line.

**Movie S2.** Dynamics of the U35 nucleobase in the "out" conformation (nucleobase oriented away from the RNA core) in the SAM-bound complexwithout Mg2+ ions.

**Movie S3.** Dynamics of M'3 during the equilibration phase of the SAM-bound simulation. The SAM ligand is displayed in ball-and-stick representation, M'3 is shown as a green sphere, the nucleobase of C25 and the phosphate groups of C27 and G28 are presented in stick representation, and water molecules are depicted in line representation.

**Movie S4.** Dynamics of M'3 during the equilibration phase of the apo-form simulation.


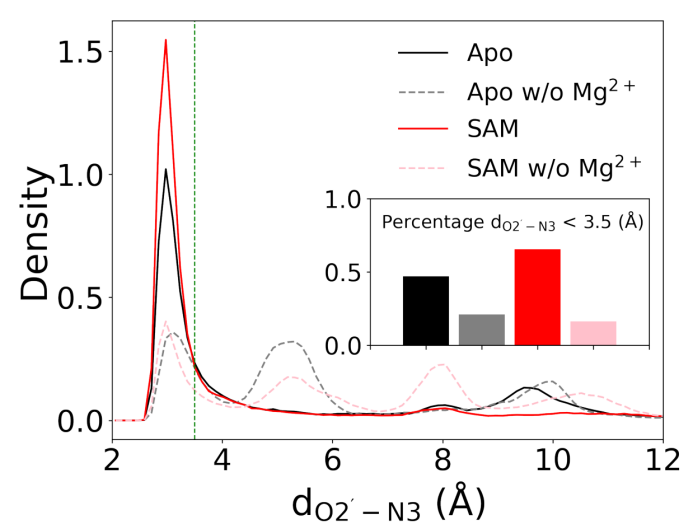


**Figure S1.** Distributions of the distance, d_O2'-N3_, between the O2' of the sugar in C24 and the N3 atom of base in U39, in the simulations for the apo form and the SAM-bound (with and without Mg^2+^). A vertical green dash line at 3.5 Å indicates the cutoff for hydrogen bond formation. Inset: fraction of frames forming this hydrogen bond.


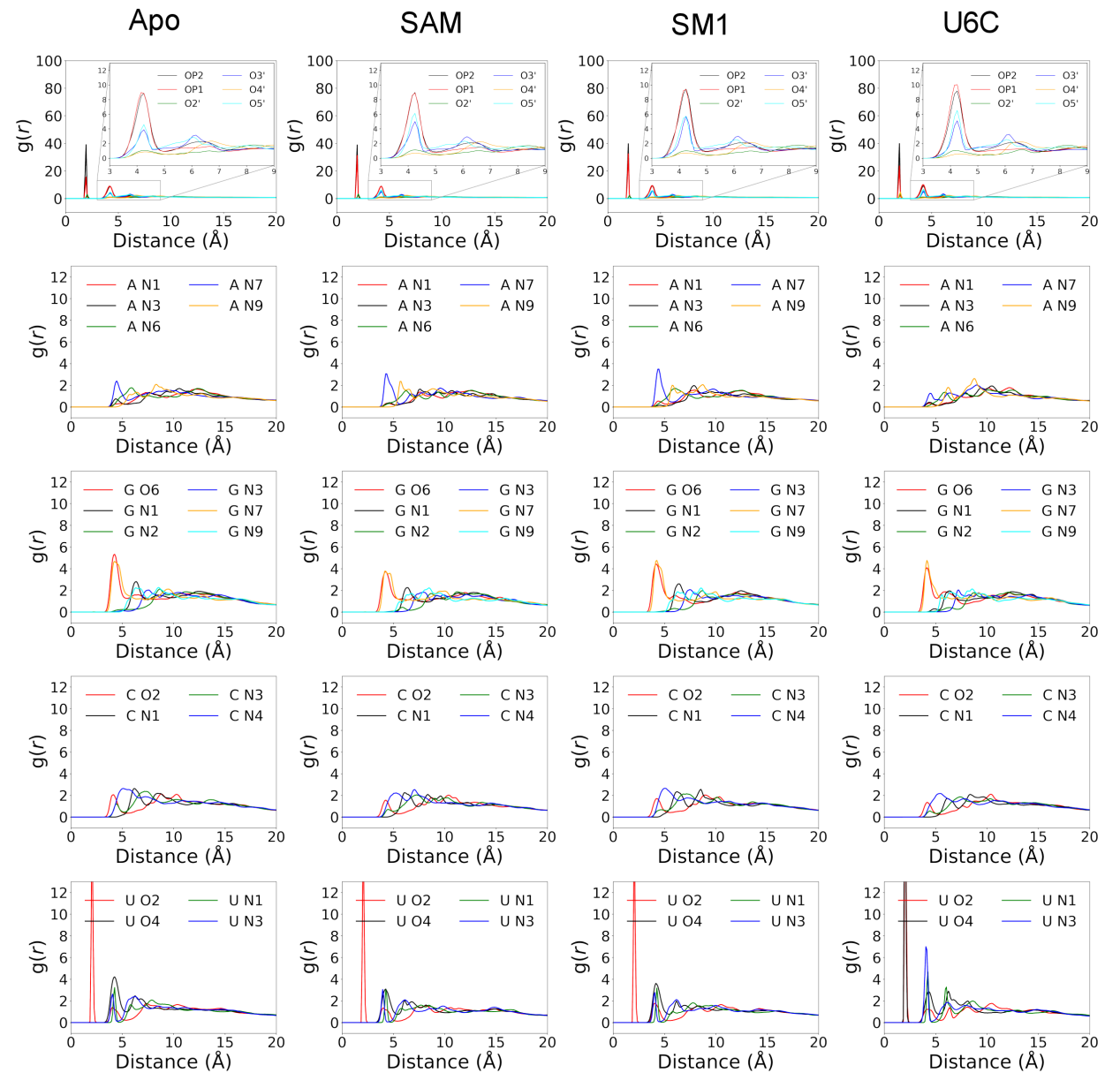


**Figure S2.** Radial distribution functions of Mg^2+^ ions around backbone and base atoms in the apo form, SAM-bound, SM1-bound, and U6C mutation..


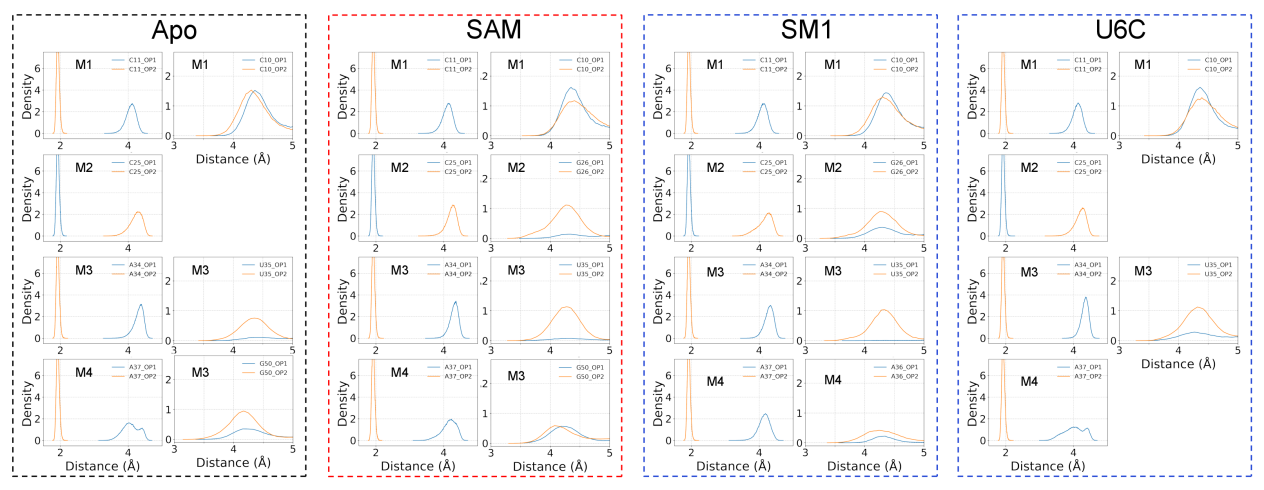


**Figure S3.** The distributions of distances between phosphate OP1 and OP2 atoms and conserved inner-shell Mg^2+^ ions in apo form, SAM-bound form, SM1-bound form, and U6C mutation.


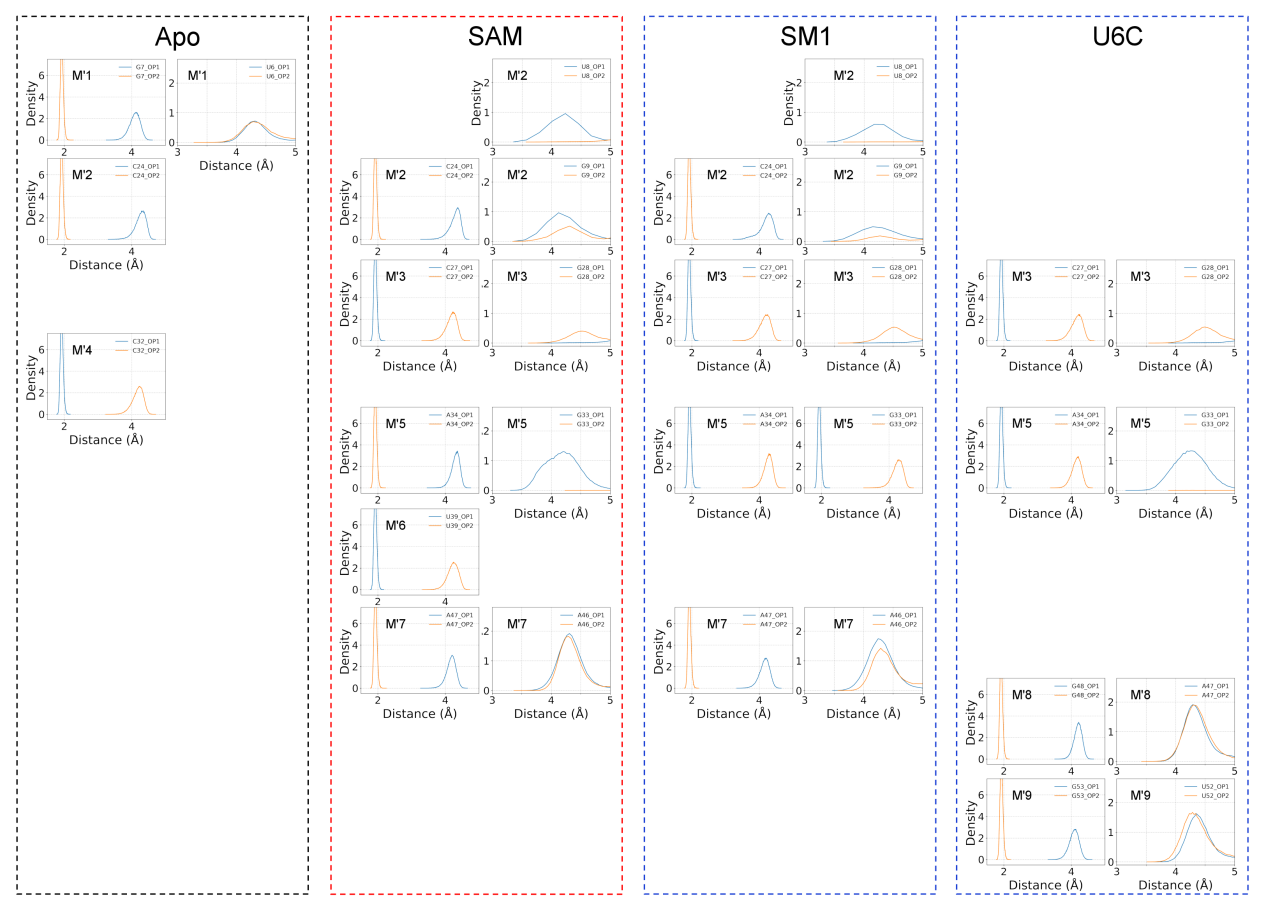


**Figure S4.** The distributions of distances between phosphate OP1 and OP2 atoms and inner-shell Mg^2+^ ions showing differences among apo form, SAM-bound form, SM1-bound form, and U6C mutation.


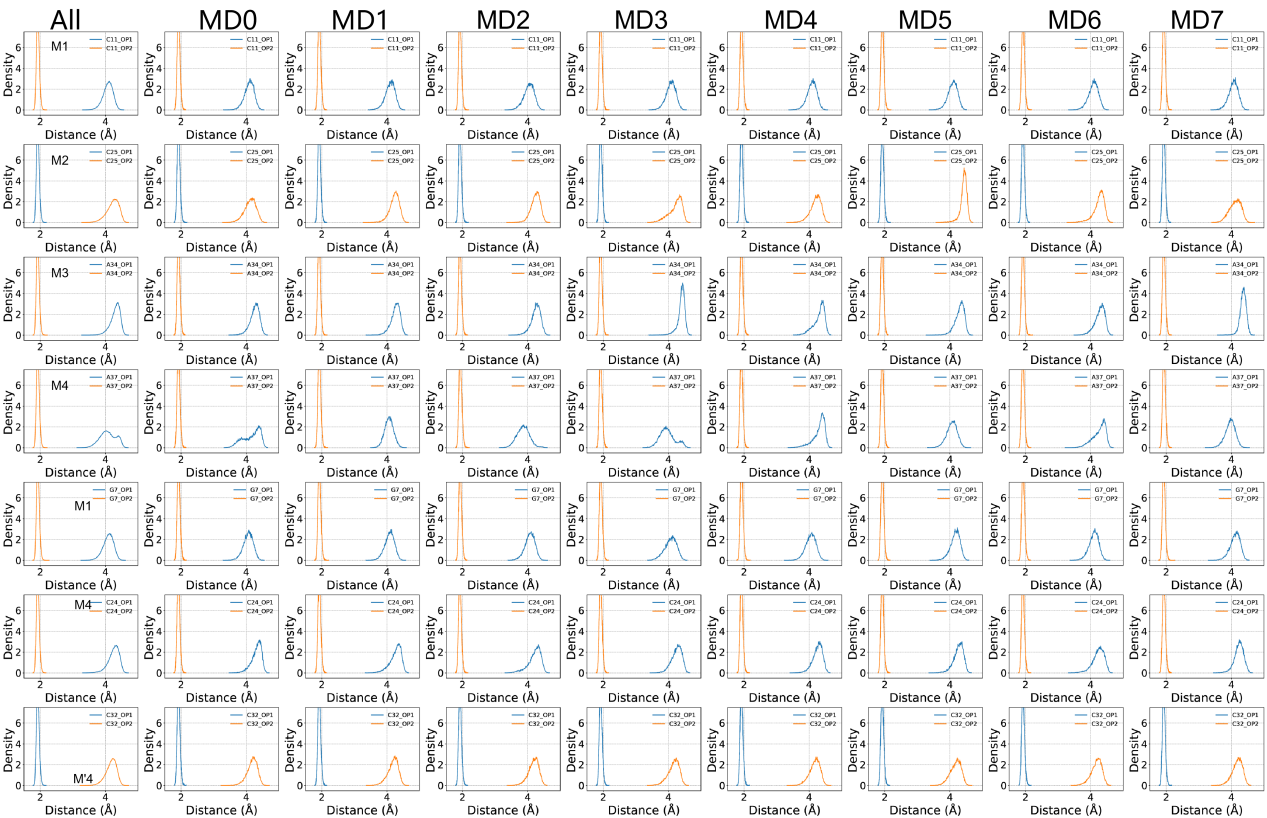


**Figure S5.** The distributions of distances between phosphate OP1 and OP2 atoms and inner-shell Mg^2+^ ions for apo form.

**
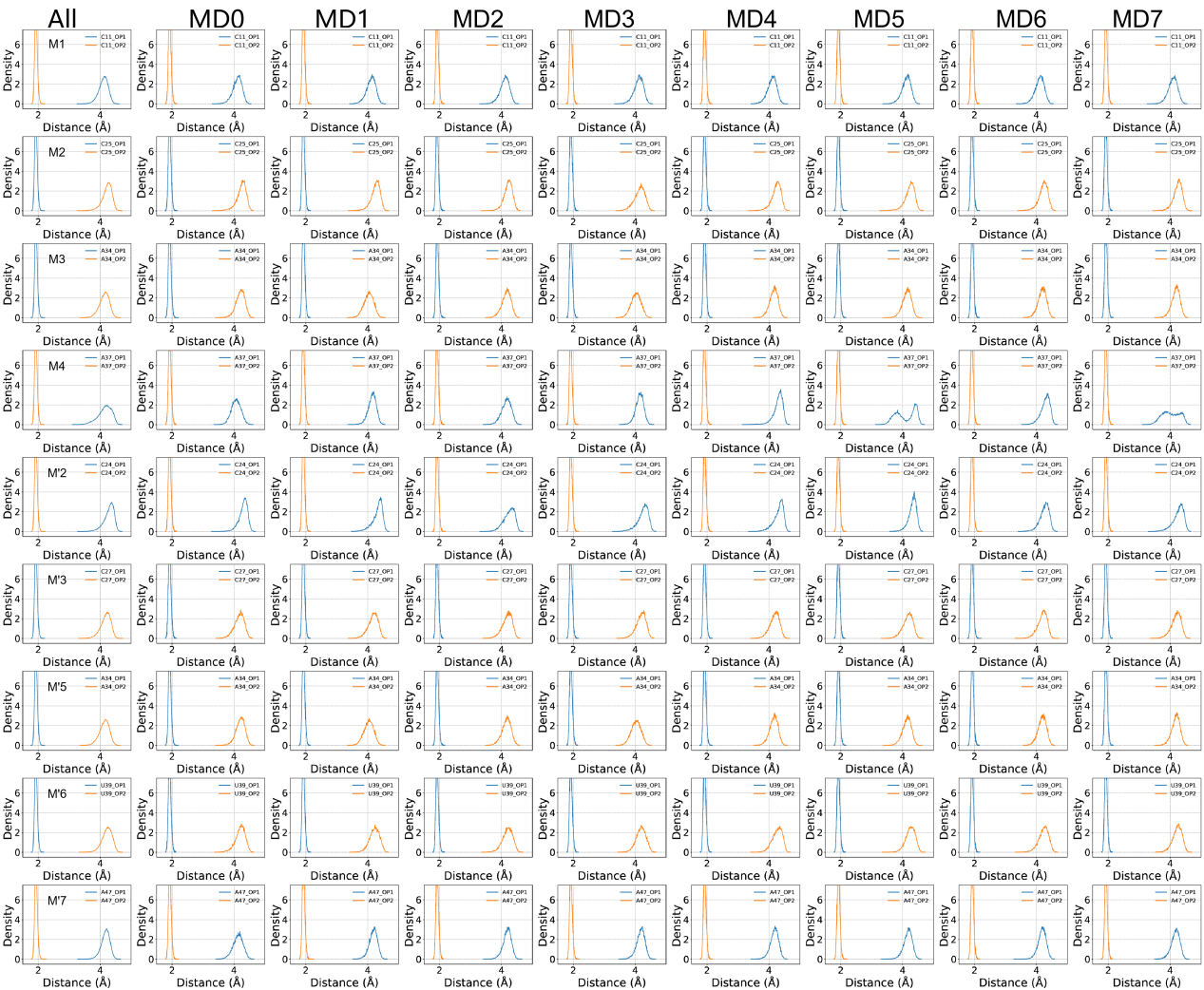
**

**Figure S6.** The distributions of distances between phosphate OP1 and OP2 atoms and inner-shell Mg^2+^ ions for SAM-bound form.

**
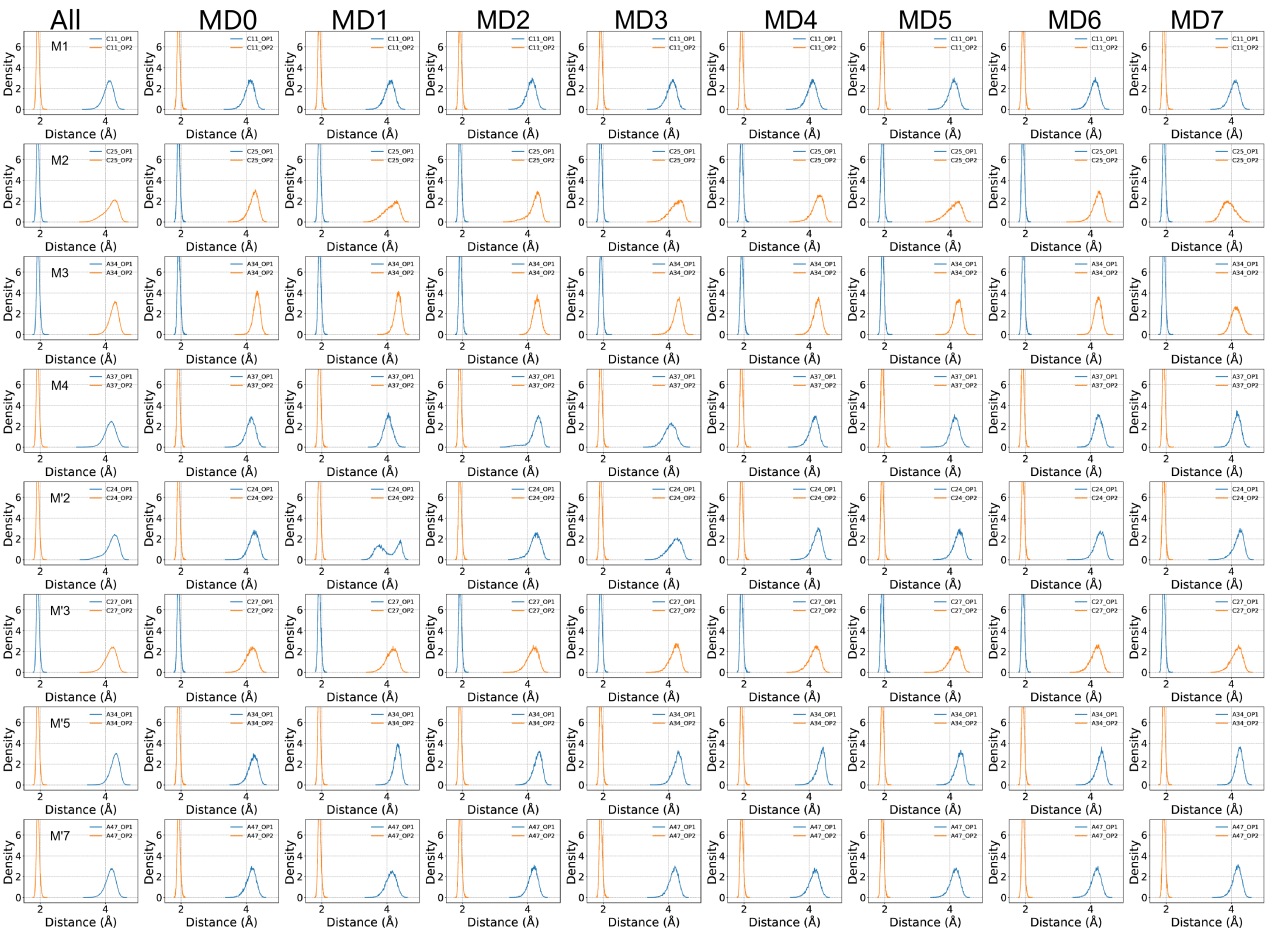
Figure S7.** The distributions of distances between phosphate OP1 and OP2 atoms and inner-shell Mg^2+^ ions for SM1-bound form.

**
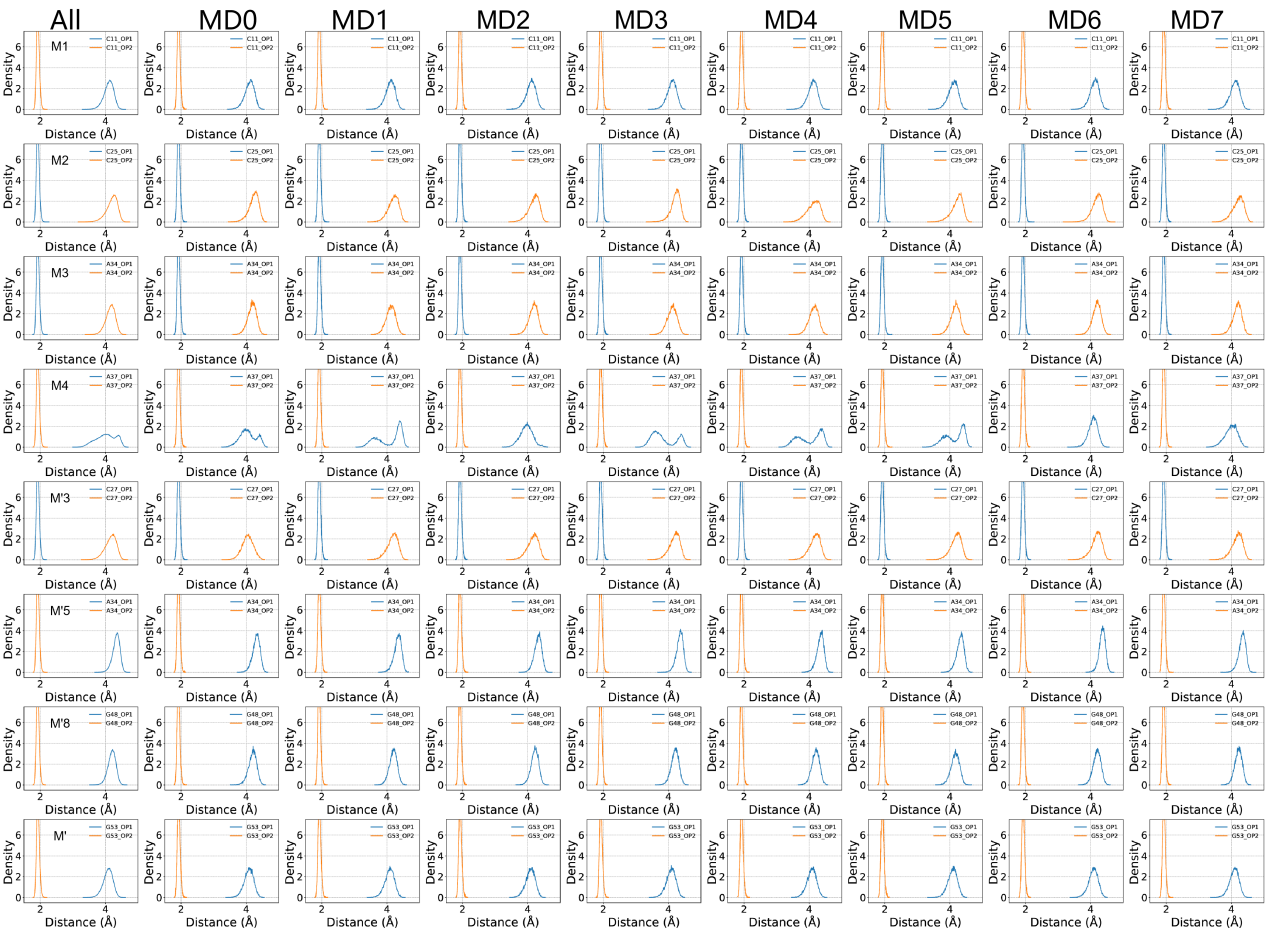
Figure S8.** The distributions of distances between phosphate OP1 and OP2 atoms and inner-shell Mg^2+^ ions for U6C mutation.


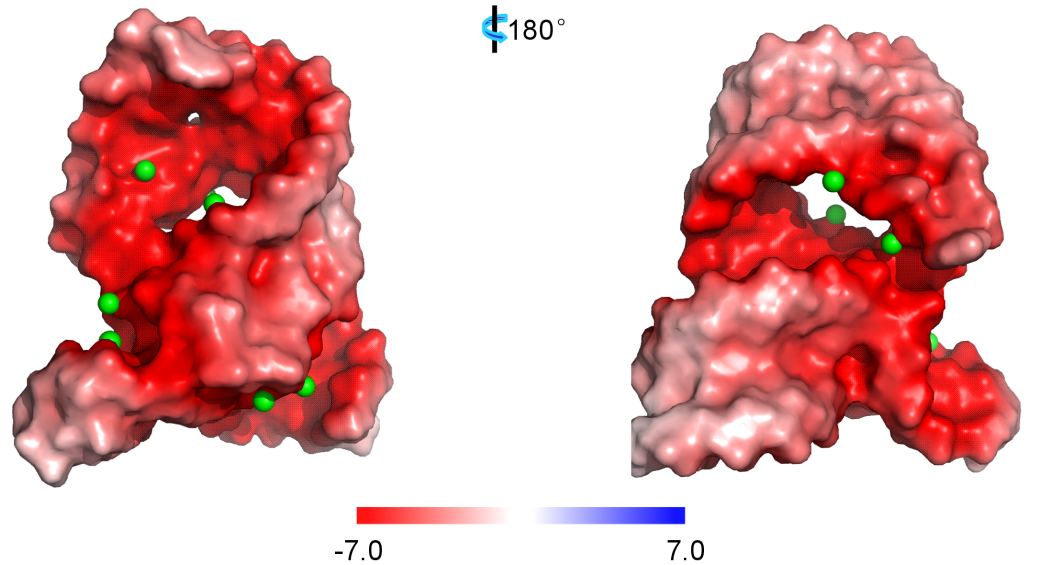


**Figure S9.** The electrostatic potential surface of the RNA, calculated using APBS [N. A. Baker, D. Sept, S. Joseph, M. J. Holst and J. A. McCammon, *Proc Natl Acad Sci U S A* 2001 **98**, 10037-10041], with a conformation derived from the MD simulations of the SAM-bound form. The ‘front’ view (left panel) and the ‘back’ view (right panel) are shown. The inner-shell Mg^2+^ ions are shown in green ball.


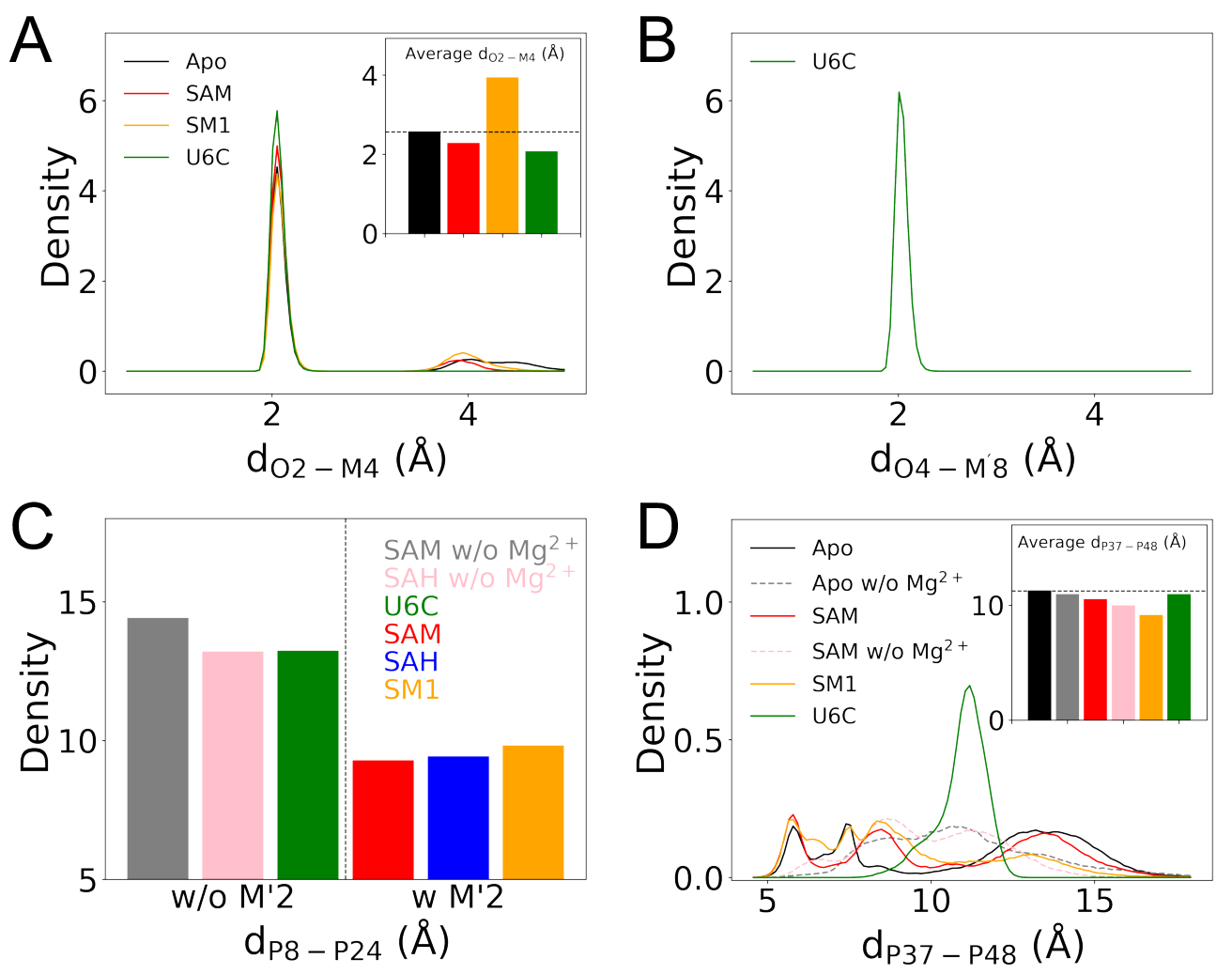


**Figure S10.** (A) Comparison of the mean distances d_P8-P24_ between the systems without (labeled as ‘w/o’) M'2 and with (labeled as ‘w’) M'2. Distributions of d_O2-M4_ (B), d_P37-P48_ (C), and d_O2-M’8_ (D). Inset: average values.


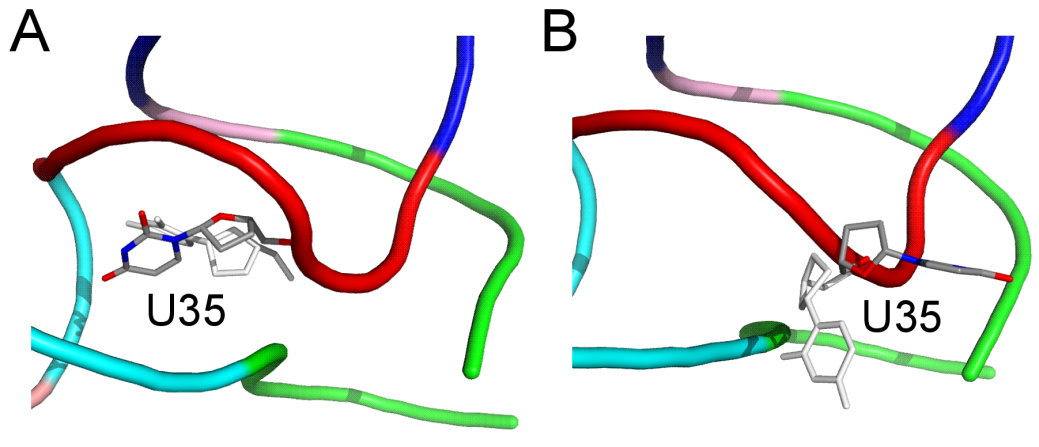


**Figure S11. The conformation of the nucleobase of U35.** (A) The “in” conformation (nucleobase oriented toward the RNA core). (B) The “out” conformation (nucleobase pointing away from the RNA core). The experimental structure (in white) and the representative structure extracted from MD simulations were superimposed by aligning the triphosphate atoms of U35, A37, and G48.


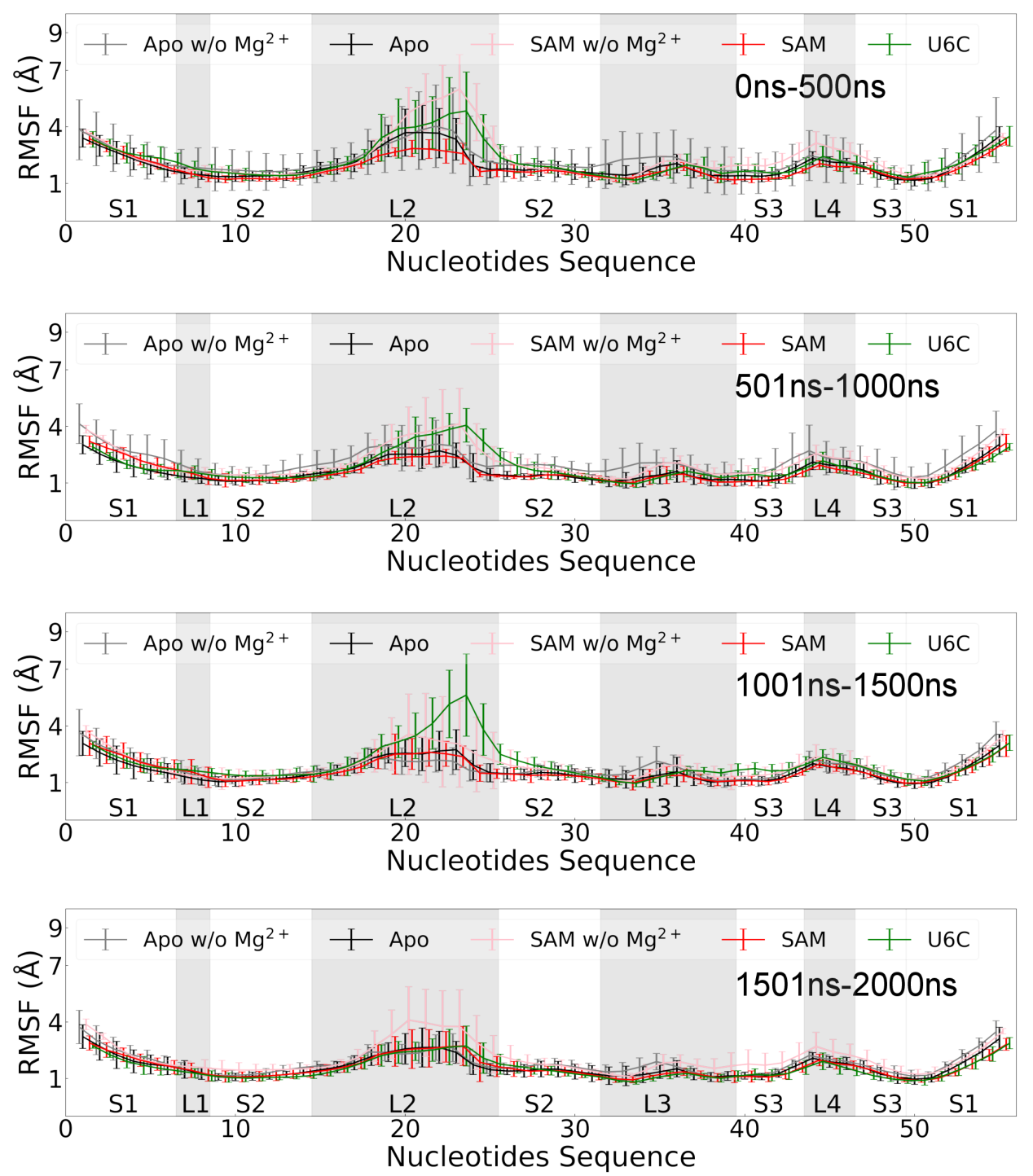


**Figure S12.** The RMSFs of P, O3', O5', C3', C4', and C5' in the apo form and SAM-bound form (with and without Mg2+), and U6C mutant. Data are averaged over eight replicate conventional molecular dynamics (cMD) runs for each 500 ns block (0-500 ns, 501-1000 ns, 1001-1500 ns, 1501-2000 ns). Error bars represent the standard deviations of RMSFs.
